# Supplementary material for: An approach in medical diagnosis based on Z-numbers soft set
Source: PLoS One. 2022 Aug 25;17(8):e0272203. doi: 10.1371/journal.pone.0272203 (PMC9409603; doi:10.1371/journal.pone.0272203)
Supplement: S1 File — (PDF) [file pone.0272203.s001.pdf]

Medical knowledgebase of Illustrative example

It should be noted that there is only a simple example in this paper with two diseases under consideration (influenza and COVID-19) to show the possibility of using this approach-based Z-numbers soft set, not a medical diagnosis in a real-world scenario.

It is quite essential to preliminary estimate by some observers or some online diagnose that the disease is more likely to be a influenza or a COVID-19 for shortage of offline outpatient service. In this paper the prim symptoms under consideration include fever (e_1), cough with chest congestion (e_2), runny nose (e_3), body ache (e_4), headache(e_5), breathing trouble(e_6), diarrhea(e_7), sore throat (e_8). To be sure the observers often provide the symptom information roughly the same as "fever will surely be very high".

There are 3 observers in this example. The judgment of the observers on the symptoms can be express by different Z-numbers soft set, in which we use converted $[0,1]$ value of degree that alternative x_i have parameter e_j and the degree of reliability. We let the universal set only contain two elements "yes" and "no", i.e. $U = \{y, n\}$, and $E = \{e_1, e_2, e_3, e_4, e_5, e_6, e_7, e_8\}$. It is given that the model Z-numbers soft sets for a influenza $\tilde{\tilde{F}}_A$ in Table 1 and a COVID-19 $\tilde{\tilde{G}}_B$ in Table 2. Now there are three related observers provide the symptom information, which can be constructed by Z-numbers soft sets $\tilde{\tilde{P}}_{O_1}$ in Table 3, $\tilde{\tilde{P}}_{O_2}$ in Table 4, and $\tilde{\tilde{P}}_{O_3}$ in Table 5.

Table 1 A ZnSS Model for influenza ($\tilde{\tilde{F}}_A$)

$\tilde{\tilde{F}}_A$	e_1	e_2	e_3	e_4	e_5	e_6	e_7	e_8
y	(0.5,0.8)	(0.5,0.8)	(0.9,0.9)	(0.2,0.8)	(0.8,0.9)	(0.2,0.8)	(0.2,0.9)	(0.8,0.9)
n	(0.5,0.8)	(0.5,0.8)	(0.1,0.9)	(0.7,0.8)	(0.2,0.8)	(0.8,0.8)	(0.8,0.9)	(0.2,0.8)

Table 2 A ZnSS Model for COVID-19 ($\tilde{\tilde{G}}_B$)

$\tilde{\tilde{G}}_B$	e_1	e_2	e_3	e_4	e_5	e_6	e_7	e_8
y	(0.9,0.9)	(0.8,0.8)	(0.1,0.9)	(0.5,0.9)	(0.5,0.9)	(0.8,0.9)	(0.5,0.9)	(0.6,0.9)
n	(0.1,0.9)	(0.1,0.7)	(0.8,0.9)	(0.5,0.9)	(0.5,0.9)	(0.1,0.9)	(0.5,0.9)	(0.4,0.9)

Table 3 A ZnSS for the ill person from observer 1($\tilde{\tilde{P}}_{O_1}$)

$\tilde{\tilde{P}}_{O_1}$	e_1	e_2	e_3	e_4	e_5	e_6	e_7	e_8
y	(0.5,0.5)	(0.4,0.8)	(0.8,0.7)	(0.2,0.8)	(0.2,0.8)	(0.5,0.5)	(0.5,0.5)	(0.6,0.5)
n	(0.5,0.8)	(0.5,0.8)	(0,1)	(0.7,0.7)	(0.6,0.7)	(0.4,0.5)	(0.4,0.5)	(0.2,0.7)

Table 4 A ZnSS for the ill person from observer 2($\tilde{\tilde{P}}_{O_2}$)

$\tilde{\tilde{P}}_{O_2}$	e_1	e_2	e_3	e_4	e_5	e_6	e_7	e_8
y	(0.8,0.8)	(0.5,0.5)	(0.5,0.7)	(0.5,0.5)	(0.5,0.5)	(0.4,0.6)	(0.2,0.8)	(0.5,0.6)
n	(0.1,0.5)	(0.5,0.6)	(0.2,0.8)	(0.5,0.5)	(0.5,0.5)	(0.4,0.7)	(0.5,0.5)	(0.3,0.6)

Table 5 A ZnSS for the ill person from observer 3($\tilde{\tilde{P}}_{O_3}$)

$\tilde{\tilde{P}}_{O_3}$	e_1	e_2	e_3	e_4	e_5	e_6	e_7	e_8
y	(0.6,0.7)	(0.2,0.5)	(0.5,0.5)	(0.4,0.7)	(0.4,0.7)	(0.5,0.6)	(0.4,0.5)	(0.6,0.5)
n	(0.1,0.8)	(0.2,0.5)	(0.3,0.6)	(0.5,0.7)	(0.4,0.7)	(0.4,0.5)	(0.4,0.6)	(0.3,0.5)
