# Supplementary material for: An approach in medical diagnosis based on Z-numbers soft set
Source: PLoS One. 2022 Aug 25;17(8):e0272203. doi: 10.1371/journal.pone.0272203 (PMC9409603; doi:10.1371/journal.pone.0272203)
Supplement: S2 File — (PDF) [file pone.0272203.s002.pdf]

## Medical knowledgebase of Comparative Example

In this paper there is only a simple example only with two diseases under consideration (influenza and COVID-19) to show the possibility of using this approach-based Z-numbers soft set, not a medical diagnosis in a real-world scenario, which is similar to the existing studies [41].

The required dataset regarding the research work in the existing studies [41] was collected from a local sub divisional Government hospital at Durgapur, West Bengal, India, after a detailed discussion about the various aspects of fever with the concerned experts. The case study in [41] is depicted based on their understanding which was gathered from the hospital. As stated in case study, after a fruitful discussion with the corresponding medical experts, a medical knowledgebase (Table 1) is designed in terms of intuitionistic fuzzy sets consisting of a set of disease and the related set of symptoms concerned with a specific disease. The diseases and symptoms are similar as defined in case study. The details of information are given in Table 1.

Table 1 Medical knowledgebase [41]

| | Viral fever | Malaria | Typhoid | Gastric ulcer | Pneumonia |
|---|---|---|---|---|---|
| Temperature | (0.6,0.3) | (0.7,0.1) | (0.4,0.1) | (0.1,0.7) | (0.1,0.8) |
| Headache | (0.6,0.1) | (0.3,0.6) | (0.3,0.1) | (0.2,0.4) | (0.2,0.5) |
| Stomach pain | (0.4,0.5) | (0.1,0.6) | (0.2,0.4) | (0.6,0.2) | (0.3,0.5) |
| Cough | (0.2,0.7) | (0.5,0.4) | (0.4,0.3) | (0.2,0.7) | (0.4,0.5) |
| Chest pain | (0.3,0.5) | (0.1,0.8) | (0.1,0.5) | (0.2,0.7) | (0.6,0.1) |
