# Supplementary material for: An approach in medical diagnosis based on Z-numbers soft set
Source: PLoS One. 2022 Aug 25;17(8):e0272203. doi: 10.1371/journal.pone.0272203 (PMC9409603; doi:10.1371/journal.pone.0272203)
Supplement: Attachment — Submitted filename: renamed_39ab2.pdf [file pone.0272203.s003.pdf]

Description of the Revisions for PONE-D-21-34053

Dear Editors and Reviewers:

We are very happy for our paper entitled "An approach in medical diagnosis based on Z-numbers soft set" has been approved to revise. We are very grateful to you and the reviewers for their valuable comments and suggestions. We have studied comments carefully and have made correction which we hope meet with approval. Revised portion are marked in yellow in the paper. Besides revised our paper according to the comments and suggestions, we also check all the definitions and theorems and replenish our paper carefully.

According to the comments given by the reviewers, we divide the comments into several parts and answered for it carefully one by one. The main corrections in the paper and the responds to the reviewer's comments are as flowing:

Reviewer #1:

This manuscript proposes a new concept of Z-number soft sets and defines some operations on Z-number soft sets, as well as a similarity measure for two Z-number soft sets. From the general perspective, the paper is interesting, however, some issues should be faced to improve the paper. There are some suggestions and comments.

Thank you very much for the suggestions and comments. We will answer for them carefully one by one.

1. The presentation quality of articles needs to be improved urgently. Some paragraphs and formats do not read well in this manuscript. There are many sentences in the article with obvious grammatical errors or unclear expressions. For example, there are three grammatical errors in the last sentence of the abstract; in subsection 2.2, the bibliography is cluttered, etc. The author should carefully check and modify.

Answer: Thank you for the comments!

Based on this comment, we have revised three grammatical errors in the last sentence of the abstract and improved the language of the whole paper carefully. For example, the tense in the abstract is changed with present. The words "presented" "gave "and "introduced" are changed into "present", "give" and "introduce", respectively. And also, for consistence, the tense of other part is changed. Our own work is mainly in the present tense. We use simple past tense when we cited the other related work. Meanwhile, we also change the other grammatical errors and unclear expressions in the other part of the paper. The details can be seen from the file named "Revised Manuscript with Track Changes".

We have check and modify the bibliography of the whole paper carefully. The details can also be seen from the file named "Revised Manuscript with Track Changes".

2. The abstract is loosely written. It is not as informative as expected. A standard abstract must present, without leaving any doubt, the objective and contribution

of the paper precisely.

Answer: Thank you for the comments!

Based on this comment, we consulted some articles in Journal PLOS ONE and rewrote the abstract which can meets PLOS ONE's style requirements. The revised abstract is condensed from five perspectives, including objectives and contributions. The details can also be seen from the file named "Revised Manuscript with Track Changes".

3. Authors should add more descriptions of motivation, both in the abstract and the introduction.

Answer: Thank you for the comments!

Based on this comment, we have added more description of motivation in the background and objective parts of the revised abstract. The details can also be seen from the file named "Revised Manuscript with Track Changes".

Meanwhile, we also have added more description of motivation in the introduction.

"One purpose of this paper is to apply the advantage of soft set to deal with uncertainty to solve this kind of special situation in medical diagnosis. For one aspect, it is free from the inadequacy of the parameterization tools of traditional mathematical tools [20]. For another aspect, theory of soft sets can be used by combining other traditional uncertainty mathematical tools." (Line 31 to Line 35 of Page 2).

"Noting that the soft set provides a technological tool to extend the concept of Zadeh's fuzzy set, while Z-numbers is a conceptual extension of a fuzzy set. As mentioned above, both of soft sets and Z-number have been widely applied to realistic areas, but the combination of Z-number and fuzzy soft set shows the theoretical applications of the two models. Motivated by the ideas of fuzzy soft sets, a direct extension would be combining soft set and Z-numbers. Therefore, in this paper, we try to conduct Z-numbers soft set to deal with nonparametric uncertainty and reliability of information by combining the advantage of the Molodtsov soft set theory and Zadeh's Z-numbers concept, which is the core purpose of this paper." (Line81 to Line 89 of Page 3).

4. A detailed proof should be given for the similarity measurement properties of two Z-number soft sets, rather than a sentence "The results trivially straightforward from definition".

Answer: Thank you for the comments!

Based on this comment, we have added the proof process (Line 349 to Line 369 of Page 10-11).

Proof. *Let* $\Delta\mu_{FG} = \left|\mu_A(x_i)_{e_j} - \mu_B(x_i)_{e_j}\right|$ *and* $\Delta\gamma_{FG} = \left|\gamma_A(x_i)_{e_j} - \gamma_B(x_i)_{e_j}\right|$ *for short.*

(1) From the definition of ZnSSs, we can have $\mu_A(x_i)_{e_j}, \mu_B(x_i)_{e_j}, \gamma_A(x_i)_{e_j}), \gamma_B(x_i)_{e_j} \in [0,1]$. *Then* $min\{\Delta\mu_{FG,}\Delta\gamma_{FG}\} \in [0,1]$ *and* $max\{\Delta\mu_{FG,}\Delta\gamma_{FG}\} \in [0,1]$ *.We can also obtain that* $1 - min\{\Delta\mu_{FG,}\Delta\gamma_{FG}\} \leq 1 + max\{\Delta\mu_{FG,}\Delta\gamma_{FG}\}$, $1 - min\{\Delta\mu_{FG,}\Delta\gamma_{FG}\} \in [0,1]$, $1 + max\{\Delta\mu_{FG,}\Delta\gamma_{FG}\} \in [1,2]$.*Therefore,* $0 \leq S(\tilde{\tilde{F}}_A, \tilde{\tilde{G}}_B) \leq 1$ *can be easily obtained.*

(2) Since $\Delta\mu_{FG} = \Delta\mu_{GF}$ *and* $\Delta\gamma_{FG} = \Delta\gamma_{GF}$, *it is obvious that* $S(\tilde{\tilde{F}}_A, \tilde{\tilde{G}}_B) = S(\tilde{\tilde{G}}_B, \tilde{\tilde{F}}_A)$.

(3) *If* $\tilde{\tilde{F}}_A = \tilde{\tilde{G}}_B$, *we have* $\mu_A(x_i)_{e_j} = \mu_B(x_i)_{e_j}$, $\gamma_A(x_i)_{e_j} = \gamma_B(x_i)_{e_j}$ *for each* $x_i \in U$.*Then we can get* $S(\tilde{\tilde{F}}_A, \tilde{\tilde{G}}_B) = 1$.

(4) *When* $\tilde{\tilde{F}}_A \subseteq \tilde{\tilde{G}}_B \subseteq \tilde{\tilde{H}}_C$, *it has* $\mu_A(x_i)_{e_j} \leq \mu_B(x_i)_{e_j} \leq \mu_C(x_i)_{e_j}$ *and* $\gamma_A(x_i)_{e_j} \geq \gamma_B(x_i)_{e_j} \geq \gamma_C(x_i)_{e_j}$ *for each* $x_i \in U$. *Then we have* $\left|\mu_A(x_i)_{e_j} - \mu_C(x_i)_{e_j}\right| \geq \left|\mu_A(x_i)_{e_j} - \mu_B(x_i)_{e_j}\right|$ *and* $\left|\gamma_A(x_i)_{e_j} - \gamma_C(x_i)_{e_j}\right| \geq \left|\gamma_A(x_i)_{e_j} - \gamma_B(x_i)_{e_j}\right|$ *for each* $x_i \in U$. *Thus* $min\left\{\left|\mu_A(x_i)_{e_j} - \mu_C(x_i)_{e_j}\right|, \left|\gamma_A(x_i)_{e_j} - \gamma_C(x_i)_{e_j}\right|\right\} \geq min\left\{\left|\mu_A(x_i)_{e_j} - \mu_B(x_i)_{e_j}\right|, \left|\gamma_A(x_i)_{e_j} - \gamma_B(x_i)_{e_j}\right|\right\}$, $max\left\{\left|\mu_A(x_i)_{e_j} - \mu_C(x_i)_{e_j}\right|, \left|\gamma_A(x_i)_{e_j} - \gamma_C(x_i)_{e_j}\right|\right\} \geq max\left\{\left|\mu_A(x_i)_{e_j} - \mu_B(x_i)_{e_j}\right|, \left|\gamma_A(x_i)_{e_j} - \gamma_B(x_i)_{e_j}\right|\right\}$. *From Operations on Z-numbers soft set, we can get* $S(\tilde{\tilde{F}}_A, \tilde{\tilde{H}}_C) \leq S(\tilde{\tilde{G}}_B, \tilde{\tilde{H}}_C)$ *easily.*

(5) *If* $\tilde{\tilde{F}}_A$ *is a crisp soft set, we have* $\tilde{\tilde{F}}_A = \{(\mu, (1,0))\}$ *or* $\tilde{\tilde{F}}_A = \{(\mu, (0,1))\}$.*Then, the complement set of* $\tilde{\tilde{F}}_A$ *can be calculated as* $\tilde{\tilde{F}}_A^C = \{(\mu, (0,1))\}$ *or* $\tilde{\tilde{F}}_A^C = \{(\mu, (1,0))\}$,*respectively. Thus,* $S(\tilde{\tilde{F}}_A, \tilde{\tilde{F}}_A^C) = 0$.

5. The article lacks a comparison with other existing studies.

Answer: Thank you for the comments!

Based on this comment, we have added a comparison with other existing studies (Line 463 to Line 503 of Page 14-15). The details can also be seen from the file named "Revised Manuscript with Track Changes".

6. The case in Section 5 is just a simple example, not a medical diagnosis in a real-world scenario, and provides little useful information in the Discussion section.

Answer: Thank you for the comments!

Based on this comment, we have explained at the beginning of section 5. (Line 411 to Line 418 of Page 12-13).

We first explain that there is only a simple example only with two diseases under consideration (influenza and COVID-19) to show the possibility of using this approach-based Z-numbers soft set, not a medical diagnosis in a real-world scenario, which is similar to the existing studies [32,41,]. We also explain the idea of applying this method in practice. On the one hand, there are not only two choices in reality to estimate for preliminary diagnosis of disease which could be improved by clinical results. In that case, the ranking results of the evaluation can help patients make the choice of registration ranking. On the other hand, the work in this paper can also help develop a platform for primary diagnosis.

In addition, we also have added a comparison with other existing studies in the

discussion (Line 463 to Line 503 of Page 14-15).

The details can also be seen from the file named "Revised Manuscript with Track Changes".

7. **Please explain the similarities and differences between the "ZnSS" in the manuscript and the "Z-set" proposed in the research "Z-set Based Approach to Control System Design".**

Answer: Thank you for the comments!21

Firstly, based on this comment, we have researched the article "Z-set Based Approach to Control System Design" and added it to the references ([67]). (Line 80 of Page 3).

Secondly, we have also added the description of similarities and differences (Line 90 to Line 96 of Page 3) between the "ZnSS" in the manuscript and the "Z-set" proposed in the research "Z-set Based Approach to Control System Design" as follows.

Both Z-set and ZnSS have a significant potential in the describing of the uncertainty of the human knowledge because it consists of restraint and reliability of the measured value in details. But ZnSS deal with nonparametric uncertainty and reliability of information by combining the advantage of the Molodtsov soft set theory and Zadeh's Z-set. In other words, ZnSS can not only measure reliability of the information effectively, but also be free from the inadequacy of the parameterization tools of traditional mathematical tools.

Finally, we also explain the similarities and differences in other relevant parts of the paper, such as in the conclusion (Line 505 to Line 510of Page 16).

Reviewer #2: The authors use the Zadeh-fuzzy number in diagnosis in medicine. The paper is solid and uses the soft set in its application. The authors should mention into the paper the extension of Soft Set to Hyper Soft Set, i.e.:

In 2018 Smarandache generalized the Soft Set to the Hyper Soft Set by transforming the classical uni-argument function F into a multi-argument function:

F. Smarandache: Extension of Soft Set to Hypersoft Set, and then to Plithogenic Hypersoft Set, Neutrosophic Sets and Systems, vol. 22, 2018, pp. 168-170. DOI: *10.5281/zenodo.2159754*

http://fs.unm.edu/NSS/ExtensionOfSoftSetToHypersoftSet.pdf

Answer: Thank you for the suggestions!

Based on this comment, we have researched the article "Extension of Soft Set to Hypersoft Set, and then to Plithogenic Hypersoft Set" and added it to the references ([31]). (Line 39 of Page 3).

Florentin Smarandache generalize the soft set to the hypersoft set by transforming the function F into a multi-attribute function, and the hybrids of Crisp, Fuzzy, Intuitionistic Fuzzy, Neutrosophic, and Plithogenic Hypersoft Set are also be introduced in that paper. Florentin Smarandache also pointed out that some applications in various engineering, medical, decision making and so on, fields of

knowledge of these types of plithogenic hypersoft sets should be investigated. These results expand the research of soft set and enrich the research field based on soft set theory.

In our paper, we focus on combing the theory of soft set and Z-numbers theory to handle the reliability of every judgment to all symptoms in details. Z-numbers were proposed by Zadeh as a new way to deal with uncertainty and reliability of information. Z-numbers can describe levels of human judgment in details. Z-numbers have a significant potential in the describing of the uncertainty of the human knowledge because it consists of restraint and reliability of the measured value in details. In this paper, therefore, we try to conduct Z-numbers soft set to deal with nonparametric uncertainty and reliability of information by combining the advantage of the Molodtsov soft set theory and Zadeh's Z-numbers concept, which is the core purpose of this paper.

Therefore, we will try to investigate these types of plithogenic hypersoft sets into the fields of medical diagnosis, decision making and so on in the future. Thank you for the suggestion again.

Reviewer #3: The paper proposed an approach to medical diagnosis and used to estimate whether two patterns or images are identical or approximately. In my opinion, this paper contains some interesting results which make a significant and technically sound contribution to the field. However, there are some issues that should be considered in the revised version.

Thank you very much for the suggestions and comments. We will answer for them carefully one by one.

1.Abstract should be restated by adding the importance and contribution of the work.

Answer: Thank you for the comments!

This comment is similar to the comment 2 given by the Reviewer #1.

We have added more descriptions of the importance and contribution of the paper precisely in the abstract. The details can be seen in the revised paper and in the answer-description of comment 2 given by the Reviewer #1.

2.Introduction section should be completely updated by adding motivation, organization and novelty of the work.

Answer: Thank you for the comments!

This comment is similar to the comment 3 given by the Reviewer #1.

We have added more descriptions of the motivation (Line14 to Line 30of Page 3, Line 47 to Line50 of Page 3), novelty (Line 97 to Line 111 of Page 3) and organization (Line 112 to Line 119 of Page 3-4) of the work in the introduction section.

The details can also be seen from the file named "Revised Manuscript with Track Changes".

3.The author should add the last two-year reference related the proposed work and link the proposed work. Please update the reference and citation.

Answer: Thank you for the comments!

Based on this comment, we have added the 7 last two-year reference (reference [5-8], [10-18],[20],[25], [28-30]) related the proposed work and link the proposed work. (Line 11 to Line 39 of Page 1). Meanwhile, we have updated the reference and citation. The details can also be seen from the file named "Revised Manuscript with Track Changes".

4.Authors should use the common symbols which can be easily understandable and readable

Answer: Thank you for the comments!

Based on this comment, common symbols are used in the revised version. The details can be seen from the file named "Revised Manuscript with Track Changes".

5.What effect is the use of the proposed model in achieving the objectives of the research? It is suggested that the results of the proposed model are stated in the conclusion with full detail.

Answer: Thank you for the comments!

Based on this comment, we have expressed the superiority of the proposed method in the conclusion with full detail (Line 518 to Line 543 of Page 16).

The advantages of the proposed method are summarized below.

(1) The traditional uncertainty mathematical theories have developed greatly and achievements have been widely applied in medical diagnosis. However, the traditional uncertainty mathematical theories have their intrinsic difficulties, which are pointed by Molodtsov[19]. Soft set theory proposed by Molodtsov has been regarded as an effective mathematical tool to deal with uncertainty.

The method presented in this paper based fuzzy extensions of soft set theory are presented can express different fuzziness of medical diagnose parameters effectively.

(2) Fuzzy numbers have been widely applied in decision making of medical diagnose. However, we found that the reliability of uncertainty symptoms in medical diagnose environments is also important. To solve this situation, Z-numbers are used to model and describe the diagnoses of decision-makers on uncertainty symptoms. Z-numbers combined with the constraint and reliability.

(3) When applying Z-numbers, we need an appropriate method for express different fuzziness of medical diagnose parameters and handle the reliability effectively. To address these problems, we combine the soft set and Z-numbers, we propose the notion of the Z-numbers soft set and treat the Z-numbers soft set as a whole, rather than converting the second component, to avoid the loss of symptoms information.

(4) Similarity measure have extensive application in the area of disease recognition. Considering the reliability of the information involved in the process, a measure of similarity between two ZnSS has been given in this paper to compare two Z-numbers soft sets in consequence, which can solve the calculation on similarities to help the final diagnose result.

(5) In the real the diagnoses of decision-making problems, the proposed method can obtain reasonable and effective results, as demonstrated by comparing the obtained

results with those from the existing methods. This method can also be applied to other multi-attribute decision-making problems.

6.In addition to expressing the superiority of the proposed method, its challenges need to be addressed.

Answer: Thank you for the comments!

Based on this comment, we have added the challenges of the proposed method in the conclusion. (Line 547 to Line 560 of Page 16-17).

"Although the proposed approach has been demonstrated to be effective through illustrative examples and in-depth discussion, there are still some aspects and potential areas that can be improved in future studies. The difference in importance between two components of a Z-number in Z-number soft set, namely the assessment value and the reliability measure need to be studied further. In this paper, determine the weight of the two components remains an unresolved issue, though the importance of these two components should be different. Second, in the proposed method for compare two Z-numbers soft set using similarity measure without considering the association between parameters. Third, the feasibility and effectiveness of the method are just verified by numerical examples rather than real professional medical knowledge in this paper. Therefore, the future study directions will include the parameterization reduction of Z-numbers soft sets. It is also desirable to further explore the applications of using the Z-numbers soft sets approach to solve real world specific problems in the process of decision making in medical diagnosis."

7.In the research methodology section, explain why this idea was proposed and what is its superiority over other methods?

Answer: Thank you for the comments!

Based on this comment, we have added explain why this idea was proposed and what is its superiority over other methods in the research methodology section. (Line 299 to Line 310 of Page 9).

"The majority of the decision-making process assumes that the decision maker's cognition for all aspects of a problem is the same. However, this is not quite true because of decision maker's inadequate experience, lack of knowledge, different risk preferences and so on. Therefore, it is quite essential to recognize the impact of the decision maker's cognition on the reliability of the information provided. We now develop an approach to solve this kind problem considering the reliability of fuzziness of problem parameters. For given objects(diseases) with certain attributes(symptoms), the Z-numbers soft set as a novel type of soft set can describe the uncertainty that not all the objects satisfy all attributes, but can show details cognitive information of one object satisfying the attribute. Because of the complexity of the real medical diagnosis, using only one aspect of information to describe uncertain events is fully difficult. A new decision-making method with new perspective is presented based on Z-numbers soft set to solve such practical decision-making problems in this section."

8.In the related works section, new resources will be used and implicitly referred

to the challenges of previous methods.

Answer: Thank you for the comments!

Based on this comment, we have improved the relevant analysis that why new resources will be used and implicitly referred to the challenges of previous methods in the related works section.

"One of the challenges in the process of this kind medical diagnosis is about how to handle the uncertainty effectively to achieve more accurate decision-making*...However, in the process of medical diagnosis, there may be many critical diseases, where experts do not have sufficient knowledge to handle those problems. For these cases, experts may provide their opinion only about certain aspects of the disease based on the symptoms they focused and remain silent for those unknown symptoms. For this kind of situation, the traditional uncertainty mathematical theories mentioned above have their intrinsic difficulties. Therefore, one purpose of this paper is to apply the advantage of soft set to deal with uncertainty to solve this kind of special situation in medical diagnosis. For one aspect, it is free from the inadequacy of the parameterization tools of traditional mathematical tools [20]. For another aspect, theory of soft sets can be used by combining other traditional uncertainty mathematical tools." (Line 14 to Line 30 of Page 2)*

"Another challenge in the process of this kind medical diagnosis is about how to measure reliability of the information effectively to achieve more accurate decision-making...However, these methods only can give whole reliability to all attributes, without giving the measuring reliability of every judgment or information in details. Z-numbers were proposed by Zadeh as a new way to deal with uncertainty and reliability of information." (Line 47to Line 49 of Page 2)

9.It is necessary to state in the related works section, why the previous methods are not responsible for solving the research problem accurately and you need to use to a new method?

Answer: Thank you for the comments!

This comment is similar to the comment 3given by the Reviewer #1.

Based on this comment, we have added the statement in the related works section that the previous methods are not responsible for solving the research problem accurately and it need to use to a new method. The details can be seen in the revised paper and in the answer-description of comment 2 given by the Reviewer #1.

We also have added explain why this idea was proposed and what is its superiority over other methods in the research methodology section (Line 299 to Line 310 of Page 9).In addition, the contribution and originality of this study are summarized in section Introduction (Line 97 to Line 111 of Page 3-4) .

10.There are too many grammatical and writing errors. The paper needs proofreading. Such as:

(1) in "Preliminaries" section formulas must be written more clearly.

(2) citation of all references must be corrected.

(3) delete title "3.1".
(4) in page 8., why some sentences have been bolded.
(5) The titles of tables (such as Table 3,4) are not true.
(6) the title of section 4 is similar to the title of this paper. Change it.
(7) Change title 4.3
(8) the explanation of the steps of Algorithm are not very clear.
(9) after title 5, please insert some words.

Answer: Thank you for the comments!

Based on this comment, we have revised nine grammatical errors as follows.

(1) All formulas have been rewritten more clearly in "Preliminaries" section.
(2) We have corrected all the citation of all references.
(3) *We have deleted title "3.1"and "3.2".*
(4) We have modified the format of bold sentences. Because some sentences have been bolded by mistake in the first draft.
(5) We have checked and revised all the titles of tables.
(6) We have changed the title of section 4, which can be distinguished from the title of the paper.
(7) Title 4.3 was cancelled and integrated with other contents again.
(8) We redescribe the explanation of the steps of Algorithm.
(9) After title 5, we have inserted some words.

Meanwhile, we also changed the other grammatical and writing errors in the other part of the paper. The details can be seen from the file named "Revised Manuscript with Track Changes".

11.For better understand, send me your code to validate your paper.

Answer: Thank you for the comments!

Based on this comment, we have updated your submission to use the PLOS LaTeX template. *The details can be seen from the file named "Revised Manuscript of the PLOS LaTeX template".*

12.The calculations have not been analyzed.

Answer: Thank you for the comments!

Based on this comment, the analysis of calculation results is improved. The details can be seen in section the analysis of calculation results from the file named "Revised Manuscript with Track Changes". In addition, we also have added a comparison with other existing studies (Line 463 to Line 503 of Page 14-15).

13.Conclusion section should be rewritten.

Answer: Thank you for the comments!

Based on this comment, the conclusion section has been rewritten. It not only includes the effect of the proposed model in achieving the research objectives (Line 505 to Line 517of Page 16), but also add the advantages (Line 518 to Line 544 of Page 16

and challenges (Line 547 to Line 560 of Page 17-18) of the proposed method.

14.The author should enrich the references section by adding the recent following references in the paper in this field and also other types of uncertainty.

Answer: Thank you for the comments!

Based on this comment, we carefully studied the recommended references and analyzed the relationship with this paper. We also added these references (reference [5-8],[20],[25], [28-31]) in the relevant sections. The details can be seen from the file named "Revised Manuscript with Track Changes".

Thank you for considering our paper!

Best wishes,

Regards,

Haiyan Zhao

Qian Xiao

Zheng Liu

Yanhong Wang
